# Supplementary material for: A Low Dietary Quality Index in a Newly Diagnosed Inflammatory Bowel Disease Cohort: Results from a Case—Control Study
Source: Nutrients. 2025 Mar 10;17(6):958. doi: 10.3390/nu17060958 (PMC11944317; doi:10.3390/nu17060958)
Supplement: Supplementary file 1 [file nutrients-17-00958-s001.zip › nutrients-3495274-supplementary.pdf]

Supplementary figures and tables:

	DRV	Ulcerative Colitis	Crohn's disease	Control
**Protein**	15	16 (4)	16 (5)	16 (3)
**Carbohydrates**	>50	46 (7)	46 (8)	46 (7)
**Total fats**	<35	35 (5)	35 (5)	36 (5)
**Saturated fats**	<11	13 (3)	14 (3)	13 (3)

Table S1: Percentage of energy derived from macronutrients, expressed as mean percentage of total energy with SD in brackets, compared to the Dietary Reference Values (DRV)

			Ulcerative Colitis	Crohn's disease	Controls
**Energy (kcal/d)**	**Male**	Mean	2575	1711	2393
		SD	1226	684	689
	**Female**	Mean	2595	1910	2580
		SD	1237	940	1201

Table S2: Total energy consumption for IBD patients and control. The dietary recommended values (DRV) for males is 2500 kcal/d and females is 2000 kcal/d. UC patients tend to exceed the DRV, whilst CD patients do not meet the DRV.
